# A survey of cariology teaching in Australia and New Zealand

**DOI:** 10.1186/s12909-018-1176-4

**Published:** 2018-04-10

**Authors:** Sarah L. Raphael, Lyndie A. Foster Page, Matthew S. Hopcraft, Peter J. Dennison, Richard P. Widmer, R. Wendell Evans

**Affiliations:** 1 Department of Population Oral Health, Faculty of Dentistry, The University of Sydney, Westmead, Australia; 2 Department of Oral Sciences, The University of Otago, Dunedin, New Zealand; 3 Melbourne Dental School, The University of Melbourne, Carlton, Australia; 4 Public Health Dentistry Specialist, Wellington, New Zealand; 5 Department of Dentistry, Children’s Hospital at Westmead, Westmead, Australia

**Keywords:** Cariology, Curriculum, Minimum intervention, Prevention, Education

## Abstract

**Background:**

The Australian and New Zealand chapter of the Alliance for a Cavity Free Future was launched in 2013 and one of its primary aims was to conduct a survey of the local learning and teaching of cariology in dentistry and oral health therapy programs.

**Methods:**

A questionnaire was developed using the framework of the European Organisation for Caries Research (ORCA)/Association of Dental Education in Europe (ADEE) cariology survey conducted in Europe in 2009. The questionnaire was comprised of multiple choice and open-ended questions exploring many aspects of the cariology teaching. The survey was distributed to the cariology curriculum coordinator of each of the 21 programs across Australia and New Zealand via Survey Monkey in January 2015. Simple analysis of results was carried out with frequencies and average numbers of hours collated and open-ended responses collected and compiled into tables.

**Results:**

Seventeen responses from a total of 21 programs had been received including 7 Dentistry and 10 Oral Health programs. Key findings from the survey were – one quarter of respondents indicated that cariology was identified as a specific discipline with their course and 41% had a cariology curriculum in written format. With regard to lesion detection and caries diagnosis, all of the program coordinators who responded indicated that visual/tactile methods and radiographic interpretation were recommended with ICDAS also being used by over half them. Despite all respondents teaching early caries lesion management centred on prevention and remineralisation, many taught operative intervention at an earlier stage of lesion depth than current evidence supports. Findings showed over 40% of respondents still teach operative intervention for lesions confined to enamel.

**Conclusion:**

Despite modern theoretical concepts of cariology being taught in Australia and New Zealand, they do not appear to be fully translated into clinical teaching at the present time.

**Electronic supplementary material:**

The online version of this article (10.1186/s12909-018-1176-4) contains supplementary material, which is available to authorized users.

## Background

Dental caries is one of the most prevalent chronic diseases in the world [1]. Despite substantial global public health efforts towards the prevention of this disease, epidemiological data shows that this significant health issue continues to affect a large proportion of the population – especially those from low socio-economic backgrounds [1–4]. In Australia and New Zealand, people who are eligible for public dental care, living in regional and rural areas or those with lower levels of education or from an indigenous background have greater levels of caries experience [5–7]. Whilst the prevalence of dental caries in developed countries dropped during the late twentieth century – these gains appear to have plateaued during the early 2000’s [8, 9]. Indeed, in Australia caries experience in children has increased by more than 50% since the mid-1990s [7]. The presentation of dental caries has also evolved from being a disease that largely affects children and young adults – to one that often presents later in life [1, 10, 11]. It is now not uncommon for adults who have been caries-free for their whole lives to present with debilitating root caries at a time when the burden of oral disease may be a serious risk for their overall health. Further there has been a shift in the sites affected by dental caries – from predominantly coronal smooth surfaces and pits and fissures to now include interproximal surfaces and root surfaces as major surfaces at risk [12, 13]. This changing face of dental caries has been widely studied during the past three decades and the understanding of lesion development as a continuum has never been more clearly defined and evidence-based [4, 10, 14].

It is now universally accepted that caries can be prevented and that caries lesions can be arrested and reversed [14–18]. This has led to conceptual and practical changes in how both caries the disease and the caries lesion are managed [10, 18–21]. For appropriate oral health care today, future practitioners need to be educated using an evidence-based curriculum with core cariology competencies that are well defined [22].

Recent surveys from Europe, USA and South American schools have investigated the current status of teaching and learning in cariology. They show that that there is some evidence of variation in content being taught. There still appears to be a mixture of very modern concepts in some schools but also more traditional concepts being taught in other schools [23–25] One of the major problems identified is that the more modern theoretical concepts of cariology, including the shift to a more preventive approach is not being adequately translated into clinical teaching [25]. This was highlighted in a newly developed curriculum in an Australian school where, although the aim of the curriculum was for a more preventive approach to underpin the whole undergraduate programme, there was limited success when evaluated [26].

The knowledge and goals of preventing dental caries has led to the collaboration of key dental and public health leaders in the formation of the Alliance for a Cavity Free Future (ACFF). Table 1 shows one of the ambitious but acheivable goals that ACFF is working towards. Goal 2 is centred around provoking change in the culture of teaching and learning within the dental and oral health training programs in order to ensure that the cariology curricula that inform the next generation of dental professionals reflect current knowledge. With this in mind, The Australian and New Zealand (ANZ) chapter of ACFF conducted a survey of key faculty members across Australia and New Zealand who were responsible for teaching the cariology program to students. The aims of the survey were to identify the current status of teaching and learning in cariology, identify potential gaps or disconnects and motivate ongoing curriculum improvement in the region.

**Table 1:** One of the Key Goals of the Alliance for a Cavity Free Future (ACFF)

ACFF Goal 2:	
Within 3 years of a chapter launch, 90% of dental schools and dental associations in the area should have accepted the philosophy behind the “new” approach of “caries as a continuum” in order to improve dental caries prevention and management.	

## Methods

The ANZ chapter of the ACFF developed a questionnaire to explore current cariology teaching principles and practice within the Dentistry and Oral Health/Dental Therapy programs throughout Australia and New Zealand. The questionnaire was developed using the framework of the European Organisation for Caries Research (ORCA)/Association of Dental Education in Europe (ADEE) cariology survey conducted in Europe in 2009 published by Schulte et al. in 2011 [23].

Throughout Australia and New Zealand there is a total of 17 Institutions teaching 24 courses in Dentistry, Oral Health, Dental Hygiene and Dental Therapy programs. This includes 15 Institutions in Australia and 2 in New Zealand (See Table 2 for the list of Institutions and Programs). These programs range from Post-graduate entry Dentistry degrees to Bachelor degrees in Dentistry and Oral Health and Diploma level courses in Dental Hygiene. Twenty-one courses were eligible to complete the survey. The three courses that teach Dental Hygiene were excluded from completing the questionnaire as cariology was not the focus of their program.

**Table 2:** Dentistry, oral health and dental hygiene programs in Australia and New Zealand

	Dentistry	Oral Health/Dental Therapy	Dental Hygiene
Australia			
New South Wales	The University of Sydney	The University of Sydney	
	Charles Sturt University	Charles Sturt University	
		Newcastle University	
Queensland	The University of Queensland	The University of Queensland	
	Griffith University	Central Queensland University	
	James Cook University		
South Australia	The University of Adelaide	The University of Adelaide	TAFE Gilles Plains
Victoria	The University of Melbourne	The University of Melbourne	TAFE Holmesglen
	La Trobe University	La Trobe University	Royal Melbourne Institute of Technology
Western Australia	The University of Western Australia	Curtin University	
New Zealand			
	University of Otago	University of Otago	
		Auckland University of Technology	

A letter of introduction was sent to the Deans/Heads of School of each eligible program informing them of the project and inviting their institution to participate in the survey. The questionnaire was constructed as an online survey and emailed to the cariology curriculum co-ordinator/s for each program in January 2015. Follow-up reminder emails were sent to participants who had not responded in March, May, September and November 2015 and the final data was collected in May 2016.

The online survey began with the ORCA definition of cariology:

“The scientific understanding of the aetiology, pathogenesis, prevention and clinical control or management of dental caries. Health outcomes related to dental caries are also of interest, as are other disorders of dental hard tissues, such as dental erosion.”

This was followed by a plain language statement about the research project outlining the principal researchers, the support and ethics approval and the purpose and potential significance of the study.

The survey comprised 75 questions covering a wide range of areas of cariology curriculum including: details of the course, format of the cariology curriculum, number of staff involved in teaching the cariology curriculum and their ongoing training, caries recording and detection methods used, use of radiographs in diagnosis and treatment planning, amount of theoretical, pre-clinical and clinical cariology teaching content within the curriculum and teaching of non-carious tooth tissue loss. The questionnaire was comprised of multiple choice and open-ended questions exploring many aspects of the cariology teaching. A copy of the survey is included in Additional file 1.

As the number of respondents was small, simple data analysis was carried out. Frequencies and average numbers of hours were collated and open-ended responses were collected and compiled into tables.

## Results

At the end of the data collection phase a total of 17 responses had been received from the 21 eligible programs across Australia and New Zealand (see Table 3 for a summary of the key results). This included 7 responses from a total of 10 Dentistry programs and 10 responses from a total of 11 Bachelor of Oral Health (BOH) programs, resulting in an overall response rate of 81%. Three responses were only partially completed – one from a Dentistry program and 2 from BOH programs.

**Table 3:** Summary of key results

Survey responses	Number of responses from total number of programs	17/21 (81%) programs
	Number of responses received - dentistry programs	7/10 (70%) programs
	Number of responses received - BOH/dental therapy programs	10/11 (91%) programs
	Number of partially completed surveys	3/17 (18%) surveys
Discipline	Cariology as a specific discipline within the program	4/16 (25%) of programs
Curriculum	Cariology curriculum in written format	7/17 (41%) of programs
Staff	Number of full time equivalent (FTE) staff teaching cariology	Average = 4.2 FTE Range = 1–10 FTE
Detection & Diagnosis	Detection of caries lesions - visual/tactile method	16/16 (100%) programs
	Detection of caries lesions - radiographic interpretation	16/16 (100%) programs
	Detection and assessment system - ICDAS recommended	10/16 (62%) programs
	Bitewing radiography as a routine component of examination	11/16 (69%) programs
Operative intervention	Cavitation as the criteria for operative intervention of caries lesion	6/16 (37%) programs
Non-Carious tissue loss	Non-carious loss of tooth tissue included in curriculum	14/14 (100%) programs
Education & Calibration	Education for clinical teaching staff in cariology	7/14 (50%) programs
	Calibration of clinical teaching staff in cariology teaching	5/14 (36%) programs

One quarter of respondents indicated that cariology was identified as a specific discipline within their institution with 7 out of the 17 (41%) stating they had a cariology curriculum in written format. With regard to which disciplines or departments carried the responsibility for teaching and learning in cariology there was a great variation in responses. Most commonly the responsibility was carried by the Conservative/Operative/Restorative Dentistry department, the Preventive/Community/Public Health department or the Paediatric Dentistry department. There was also a wide variation in the number of full time equivalent (FTE) staff members responsible for cariology teaching – ranging from 1 to 10 FTE staff with an average of 4.2.

**Fig. 1:**
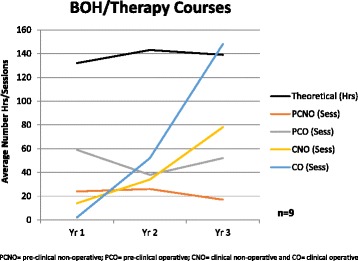
Average number of hours/sessions of cariology teaching in BOH/Dental Therapy Courses

**Fig. 2:**
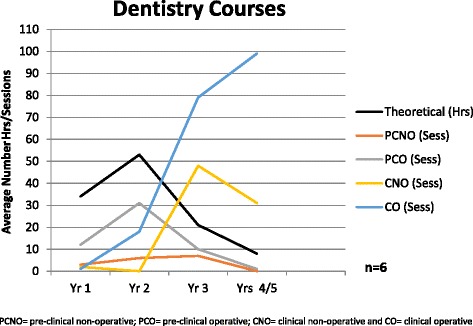
Average number of hours/sessions of cariology teaching in Dentistry Courses

Within Australian and New Zealand programs, the cariology curriculum is delivered in theoretical, pre-clinical and clinical courses throughout the years of the Oral Health/Dental Therapy and Dentistry programs. Pre-clinical and Clinical experience was further stratified into non-operative (including behaviour management, oral health education, fissure sealing and fluoride applications) and operative (restoration) for the purposes of the survey. Feedback from respondents indicated that the questions relating to the number of hours devoted to the delivery of the curriculum were cumbersome and difficult to complete accurately. However, the final data received in this section included responses from 9 BOH/Dental Therapy programs (Fig. 1) and 6 Dentistry programs (Fig. 2) with theoretical teaching recorded in hours and pre-clinical and clinical teaching recorded in sessions (of 2–3 h duration).

Figure 1 shows that in the BOH/Dental Therapy programs the number of hours of theoretical teaching in cariology remains at a constant level throughout the three-year program. The number of pre-clinical non-operative (PCNO) and pre-clinical operative (PCO) sessions peak during Year 2 of the three-year program. With regard to clinical sessions – including both non-operative (CNO) and operative (CO), the number of sessions increases from Year 1 through to Year 3. The results of the survey also show that CNO experience accounts for a greater proportion of clinical time in Year 1 and 2 of the program compared with Year 3. During the final year of their program (Year 3) students spent approximately one third of their clinical cariology experience in non-operative sessions compared with two thirds in operative sessions (Fig. 1).

For the Dentistry Courses, Years 4 and 5 are combined in Fig. 2 as some courses are of 4-year duration and others of 5-year duration. The results of the survey show that the number of hours of theoretical teaching of the cariology curriculum peaks in Year 2 of the program. Fig. 2 also shows that PCNO and PCO sessions occur mainly in the first half of the program and CNO sessions peak during the third years of the program. In the final years, students spent approximately one quarter of their clinical cariology experience in CNO sessions compared with three quarters in CO sessions.

**Fig. 3:**
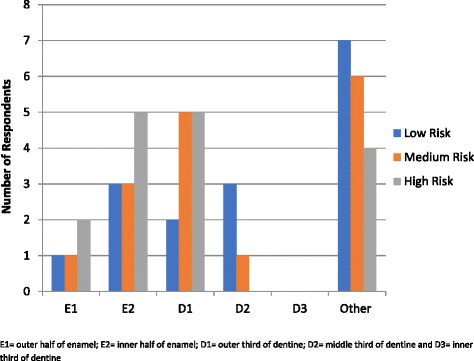
Radiographic Threshold for Operative Intervention – Primary Dentition

**Fig. 4:**
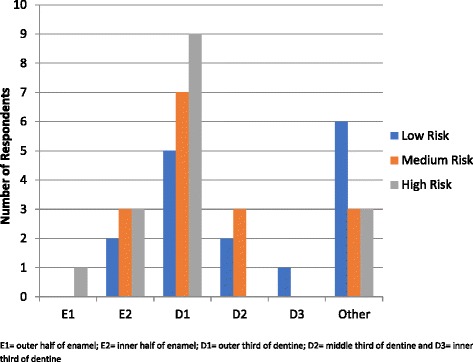
Radiographic Threshold for Operative Intervention – Permanent Dentition

Responses to the question on the radiographic threshold which indicates a need for operative intervention in patients of low, medium and high caries risk in the primary and permanent dentitions are shown in Figs. 3 and 4 respectively.

When considering the primary dentition and associated caries risk of the patient (low, medium and high) nearly half of schools (43%) teach operative intervention for lesions confined to enamel with a greater proportion recommending this for high risk patients (58%) than low risk patients (44%) as shown in Fig. 3. For the permanent dentition (Fig. 4), a quarter of schools (25%) teach operative intervention for lesions confined to enamel and the same trend exists with a greater proportion recommending this for high risk patients (30%) than low risk patients (20%).

With regard to the detection of caries lesions and the diagnosis of dental caries, all respondents indicated that visual/tactile and radiographic interpretation were the most commonly used methods – with ICDAS also being frequently recommended. Although respondents from all programs answered that the use of bitewing radiography for both adult and child patients was recommended to be used as a diagnostic tool for patients based on their risk assessment rather than at any set interval of time, 11 out of 16 (69%) program co-ordinators indicated that the rationale for taking bitewing radiographs was that it formed a routine component of the examination.

In relation to the question on radiographic indication for operative intervention, many schools selected the “Other” response on the basis that decision-making could not be defined by radiographic depth of the lesions. The central theme of these “Other” responses summarised in Table 4 was that various other clinical and risk factors needed to be considered rather than being able to make the decision solely on radiographic signs.

Six courses identified cavitation as the visual/tactile severity stage at which operative intervention is required, but four of these same courses recommended operative intervention when radiography showed lesions in the enamel or outer third of the dentine.

With regard to non-carious loss of tooth tissue the survey showed that 100% of those who answered, indicated that erosion, abrasion and attrition of dental hard tissues are included in the curricula. There were varied responses to the year levels in which these topics were taught but it appeared that many programs covered these topics throughout the earlier years.

The questionnaire also explored the topic of education for part-time and casual staff as well as members of faculty to ensure consistent application of teaching philosophies in the clinic. Results from these questions showed that only half of the programs provided training or education programs aimed towards this goal. Furthermore, two thirds of programs responded that they did not offer calibration exercises to ensure consistency of teaching in clinical cariology.

This survey explored key aspects of the cariology curriculum of each program. Table 3 lists a summary of the principal areas of interest. A small number of open-ended questions from the survey have not been reported due to the diverse nature of the answers and the difficulty in summarising the information succinctly.

## Discussion

This survey, which is the first of its kind undertaken in Australia and New Zealand, has provided some invaluable insights into the teaching and learning practices of cariology in this region. The results confirm the importance of cariology within the overall structure of the Dentistry and Oral Health curricula. Although the number of respondents is small – due to the low number of dental and oral health schools in Australasia, the survey has identified a key disconnect between the theoretical philosophy and the practical application of cariology teaching in the region.

In only one quarter of schools was cariology identified as a specific discipline, and it was predominantly taught in Restorative/Conservative Dentistry or Paediatric Dentistry Departments. This matches the international experience, with less than one-fifth (16.7%) of Latin American Schools having a separate Department of Cariology [24]. A low number of teaching programs had the cariology curriculum available in written format which may highlight the lack of importance the teaching and learning of cariology has in Australasian programs. Following a similar survey in European dental schools it was recognised that there was a need for students to receive a systematic education in cariology and a set of core European cariology competencies were defined in 2011 [22]. The European Core Curriculum in Cariology document outlined broad competencies (theoretical, pre-clinical and clinical stages) for consistency of content within European programs [22]. Other existing international efforts towards a core curriculum for cariology have also aimed at consistency with systematic teaching efforts and a formalised written curriculum [24, 27, 28].

The Australian Dental Council is responsible for accrediting dental programs in Australia, and has developed professional competencies for newly graduated dental practitioners in consultation with the dental profession as a point of reference for accreditation [29]. These competencies talk only in general terms about disease management, prevention and early intervention, and there is no specific guidance on what cariology competencies should be. As a consequence, schools are operating in a vacuum.

Although the didactic teaching of cariology is predominantly delivered by academic staff, the translation from theory to preclinical and clinical application of that knowledge is mainly supervised by part-time casual supervisors. The education for these part-time and casual staff has been shown to be limited, with only half of the programs providing training for these staff members and one third offered calibration exercises. These part-time and casual staff often work predominantly in private practice, and in many cases their training in cariology was significantly different from the approach that is now being taken in dental schools. The lack of training and calibration noted in the present study is likely to have a large effect on the education program [25]. Fontana and Zero [25], found that phrases such as “this is not real-life dentistry” or “you will never have to do this in practice” may be encountered by student clinicians and are therefore likely to undermine their cariology learning. It is not surprising to find that in many Australasian schools, clinical management of caries lesions in teaching clinics is not aligned with current understanding and teaching of the pathogenesis of the disease. Education of the teaching faculty (‘teaching the teachers’) should receive greater emphasis to align and reduce existing variations in the teaching staff.

Whilst it appears that the didactic component of cariology is well taught in the majority of Australasian programs, there is a significant disconnect in the clinical application of that teaching. In the majority of the Australasian programs, the responsibility for teaching the surgical and non-surgical management of caries lesions lies within different departments (restorative dentistry and preventive dentistry). This situation leads to an assumption from both students and staff that effective non-surgical management of caries lesions is completely separate from restorative treatment planning. This concept is often reinforced by the value placed on restorative treatment from the clinical perspective – whether it is quotas or clinical assessment of restorative procedures, or even the ‘achievement’ of students undertaking their first restoration. It is further illustrated by the time allocation that Australian and New Zealand courses attribute to clinical-operative versus clinical non-operative sessions in the final year/s of their courses – where clinical non-operative sessions make up only one third of BOH/Therapy students sessions and approximately one quarter of Dentistry students clinical experience.

This survey showed that many of the schools were using a caries detection and assessment system that includes non-cavitated lesions, namely ICDAS. However, despite all respondents indicating they teach management of early disease, centred on lesion arrest and remineralisation, many taught operative intervention at an earlier stage of lesion depth than current evidence supports. Operative intervention for caries lesions confined to enamel is still taught in nearly half of the programs. Even within the six programs that used cavitation as the criteria for operative intervention of lesions, four of these responded that operative intervention at the inner half of enamel or outer third of dentine was recommended. This is not too dissimilar to findings from dental schools in the Latin American countries [24] and reflects a lack of adherence to the current caries paradigm shift.

Many respondents believed risk factors needed to be considered when deciding on the management of a lesion (see Table 4). Although on average, lesion progression from enamel to dentine tends to be slow, an assessment of caries risk is necessary at the outset of dental care and such care needs to follow a risk-specific protocol to arrest and remineralise non-cavitated lesions [30–33]. This survey suggests that although caries risk assessment is taught and considered in the decision-making of caries lesion management, most Australasian programs are teaching students to restore lesions in the outer third of dentine. It is clear that non-operative caries management strategies are not being implemented on a regular basis across Australia and New Zealand, although they are encouraged as part of the theoretical teaching of cariology. It is critical that this approach is supported during clinical placements within and outside the faculty clinics.

**Table 4:** Reasons given in “Other” response for radiographic threshold for operative intervention

Radiographic Threshold for Operative Intervention – Other reasons specified
We have an adjustment of the ICDAS classification as follows: For C3 lesions: Radiolucency extending beyond ADJ just into dentine (0.5 mm). At this point we teach the students that for a low risk patient with a C3 lesion is recommended to remineralise it by applying fluoride varnish. Our C4 classification is: Radiolucency with obvious spread in the outer 1/3 of dentine. If a patient is medium or high risk then intervention is recommended based on risk assessment (existing restorations, carious lesions). For Medium risk in the permanent dentition – clinical judgement should be considered. Since we are talking about adults maybe preventative treatment should be considered as the patient might change the diet and hygiene practices
Our definition of low caries risk is no active primary or secondary caries lesions. Therefore, a low caries risk patient would not require operative treatment.
For Low and Medium risk in the primary dentition - due to unreliable clinic visits in country clinic, outer 1/3 of dentine unless mesial/distal of primary 4.
For primary and permanent dentition – all risk status – intervention is according to the Caries Management System- only if cavitation detected.
For Low and Medium risk in the primary dentition and Low risk in the permanent dentition - intervention is based on age of patient, fluoride exposure, caries risk, location of lesion, oral hygiene practices, parental supervision and guidance, dexterity
For both primary and permanent dentitions – intervention is based on caries risks and other clinical findings and taught on case-by-case basis. Age of child, time to exfoliation, access to surface remineralising agents
For intervention in the primary dentition - depends on a number of different variables: A) Dependent on the ‘age’ of the tooth i.e. how long before exfoliation B) The site of the caries C) Any patterning noticed D) The length of time the lesion has taken to develop (usually gauged by looking at the case notes from the last examination etc.) E) The potential for caries arrest or at least hamper in its continued development. For Medium Risk in Primary Dentition - Would usually consider intervention once the caries has breached the DEJ by 1-2 mm For High Risk in Primary Dentition - often you are seeing lesions with these patients beyond the middle third and beyond a reversible pulpitis For Low Risk in the Permanent Dentition – it is sometimes dependant on the potential for remineralisation/caries arrest, the site, the duration of formation etc.

This study has identified some probable explanations for this disconnect between the theoretical and clinical management of caries in the Dentistry and Oral Health programs across Australia and New Zealand and will be a sound basis on which to drive ongoing curriculum improvement in the region.

The results and conclusions drawn by this study are limited by the small number of institutions across Australia and New Zealand that teach programs in Dentistry, Dental Therapy and Oral Health Therapy. However, responses were received from 17 of the total 21 programs, with good representation from both Dentistry and Dental Therapy/Oral Health Therapy. As a result, despite the small numbers, this survey has been able to give an accurate and up-to-date picture of the status of the cariology teaching in this region and allowed comparison with similar studies carried out in other parts of the world.

The primary recommendations from this survey are:

1. For Australia and New Zealand cariology educators to develop core cariology curricula and for each school or program to develop or maintain a written curriculum to inform the teaching and learning of cariology across all disciplines.
2. For each school or program to provide staff education and calibration exercises to align with current theoretical best practice and translate these into the clinical caries management.
3. For each school or program to monitor the clinical application of caries management, to ensure that those clinicians responsible for clinical supervision are not undermining the risk-assessment and preventive caries management approach being taught didactically and that the spectrum of caries management be brought under the umbrella of a single department or unit within schools to ensure that restorative intervention is not over-emphasised later in the curriculum.
4. To conduct a follow-up survey in the future to track the progress in achieving the goal of curriculum improvement and translational learning in cariology ultimately leading to the development of a consensus cariology curriculum implemented throughout the Dentistry and Oral Health teaching and learning programs in Australia and New Zealand.

## Conclusion

Despite modern theoretical concepts of cariology being taught in Australia and New Zealand, they do not appear to be fully translated into clinical teaching at the present time. Four primary recommendations to motivate ongoing cariology curriculum improvement and improve translation into the clinical application of caries management have been made.

## Additional file

Additional file 1: Cariology curriculum survey for Australia and New Zealand. (PDF 199 kb)

## Abbreviations

%: Percentage
ACFF: The Alliance for a Cavity Free Future
ADEE: Association of Dental Education in Europe
ADJ: Amelodentinal junction
ANZ: Australian and New Zealand
BOH: Bachelor of Oral Health
CNO: Clinical non-operative
CO: Clinical operative
DEJ: Dentino-enamel junction
FTE: Full time equivalent
ICDAS: International caries detection and assessment system
ORCA: European Organisation for Caries Research
PCNO: Pre-clinical non-operative
PCO: Pre-clinical operative
