# Supplementary material for: A survey of cariology teaching in Australia and New Zealand
Source: BMC Med Educ. 2018 Apr 10;18:75. doi: 10.1186/s12909-018-1176-4 (PMC5892021; doi:10.1186/s12909-018-1176-4)
Supplement: Supplementary file 1 — Cariology curriculum survey for Australia and New Zealand. (PDF 199 kb) [file 12909_2018_1176_MOESM1_ESM.pdf]

Cariology can be defined as “the scientific understanding of the aetiology, pathogenesis, prevention and clinical control or management of dental caries. Health outcomes related to dental caries are also of interest, as are other disorders of dental hard tissues, such as dental erosion.” (European Association for Caries Research)

Plain Language Statement

You are invited to participate in a research project which is being conducted by A/Prof Wendel Evans (University of Sydney), Dr Sarah Raphael (Colgate) and A/Prof Matthew Hopcraft (University of Melbourne).
This project is being supported by Colgate and the Alliance for a Cavity Free Future (www.allianceforacavityfreefuture.com.au), and has been approved by The University of Sydney Human Research Ethics Committee.

Purpose of the Study
The aim of this study is to establish a database covering learning and teaching in cariology in dentistry, oral health therapy, dental hygiene, and dental therapy schools in Australia, New Zealand, Fiji, and Papua New Guinea. This study requires dental academics involved in the teaching of cariology to complete a short online questionnaire. This should take approximately 20-30 minutes to complete.

Potential significance of the study
The results will highlight the current status of cariology teaching and learning in this region and serve to motivate ongoing curriculum improvement.

1. Which program are you responsible for teaching Cariology in?

- ◯ Dentistry
- ◯ Oral health/dental hygiene/dental therapy
- ◯ Both

* 2. If your school delivers both Dentistry and Oral Health programs, are students in each program exposed to the same the Cariology curriculum.

- ◯ No
- ◯ Yes, for all components
- ◯ Yes, for some components
- ◯ Other (please specify)

* 3. At your dental school, is Cariology identified as a specific discipline?

- ◯ Yes
- ◯ No

* 4. Is the Cariology curriculum available in a written format?

- ◯ Yes
- ◯ No

5. Which of the following disciplines/departments at your school have specific responsibilities for learning and teaching in Cariology? (Identify all that are applicable)
If your school is not associated with a dental school and has, for example a Hygiene stream and/or a Therapy stream, please select ‘Dental Therapy’ or 'Dental Hygiene' as appropriate.

- ☐ Paediatric Dentistry
- ☐ Conservative/Operative/Restorative Dentistry
- ☐ Preventive Dentistry/Community Dentistry/Public Health Dentistry
- ☐ Microbiology
- ☐ Biochemistry
- ☐ Oral Pathology
- ☐ Dental Therapy
- ☐ Dental Hygiene
- ☐ Other (please specify)

6. How many staff (FTEs) are responsible for teaching Cariology in your dental program?

(FTE staff)

* 7. Which Cariology textbooks are recommended for Cariology teaching at your school?

* 8. Which caries detection methods are recommended at your school? (select all that apply)

- [ ] ICDAS
- [ ] Visual/tactile techniques
- [ ] Radiograph interpretation
- [ ] DMFT
- [ ] Fluorescence based methods (ie. DiagnoDent)
- [ ] Orthodontic separators
- [ ] Other (please specify)

* 9. For adult patients, when do you teach that bitewing radiographs should be taken for patients?

- ( ) Every 6 months
- ( ) Every 12 months
- ( ) Every 24 months
- ( ) Based on a risk assessment
- ( ) Other (please specify)

* 10. For child patients, when do you teach that bitewing radiographs should be taken for patients?

- ( ) Every 6 months
- ( ) Every 12 months
- ( ) Every 24 months
- ( ) Based on a risk assessment
- ( ) Other (please specify)

* 11. What is the rationale for taking bitewing radiographs that you teach students? (select all that apply)

- [ ] Confirm visual/tactile diagnosis
- [ ] If patients are considered high caries risk
- [ ] Monitoring carious lesions
- [ ] Routine component of the examination
- [ ] Prior to placement of fissure sealant
- [ ] Other (please specify)

* 12. At what radiographic depth of the lesion do you teach that operative intervention is required for LOW RISK patients and PRIMARY TEETH? (tick only one box)

- Outer half of enamel
- Inner half of enamel
- Outer third of dentine
- Middle third of dentine
- Inner third of dentine
- Other (please specify)

* 13. At what radiographic depth of the lesion do you teach that operative intervention is required for MEDIUM RISK patients and PRIMARY TEETH? (tick only one box)

- Outer half of enamel
- Inner half of enamel
- Outer third of dentine
- Middle third of dentine
- Inner third of dentine
- Other (please specify)

* 14. At what radiographic depth of the lesion do you teach that operative intervention is required for HIGH RISK patients and PRIMARY TEETH? (tick only one box)

- Outer half of enamel
- Inner half of enamel
- Outer third of dentine
- Middle third of dentine
- Inner third of dentine
- Other (please specify)

* 15. At what radiographic depth of the lesion do you teach that operative intervention is required for LOW RISK patients and PERMANENT TEETH? (tick only one box)

- ◯ Outer half of enamel
- ◯ Inner half of enamel
- ◯ Outer third of dentine
- ◯ Middle third of dentine
- ◯ Inner third of dentine
- ◯ Other (please specify)

* 16. At what radiographic depth of the lesion do you teach that operative intervention is required for MEDIUM RISK patients and PERMANENT TEETH? (tick only one box)

- ◯ Outer half of enamel
- ◯ Inner half of enamel
- ◯ Outer third of dentine
- ◯ Middle third of dentine
- ◯ Inner third of dentine
- ◯ Other (please specify)

* 17. At what radiographic depth of the lesion do you teach that operative intervention is required for HIGH RISK patients and PERMANENT TEETH? (tick only one box)

- ◯ Outer half of enamel
- ◯ Inner half of enamel
- ◯ Outer third of dentine
- ◯ Middle third of dentine
- ◯ Inner third of dentine
- ◯ Other (please specify)

* 18. At which clinical visual/tactile severity stage of the lesion do you teach that operative intervention is required? (tick only one box)

- ◯ Distinct cavity with visible dentine
- ◯ Underlying shadow/opacity
- ◯ Micro-cavity/enamel breakdown
- ◯ White/brown spot lesion
- ◯ Other (please specify)

* 19. Is a risk management approach to caries management taught at your school?

- ◯ Yes
- ◯ No

20. If yes, which risk factors are considered in caries risk management? (tick all that apply)

- [ ] Past caries history
- [ ] Plaque/oral hygiene
- [ ] Newly erupted teeth
- [ ] Exposed root surfaces
- [ ] Medication related to mouth dryness
- [ ] Related diseases
- [ ] Bacteria (Lactobacillus/S.mutans)
- [ ] Fluoride exposure (professional, self-care, water fluoridation)
- [ ] Diet (contents)
- [ ] Diet (frequency)
- [ ] Saliva
- [ ] Lifestyle – drug use
- [ ] Other (please specify)

* 21. Is early caries management (caries prevention, remineralisation) taught at your school?

- ◯ Yes
- ◯ No

22. If early caries management (caries prevention, remineralisation) is taught, please indicate which topics are considered? (select all that apply)

- [ ] Professional plaque removal
- [ ] Oral hygiene instruction
- [ ] Cariogenic diet management
- [ ] Professional fluoride application
- [ ] Home fluoride supplement use (mouthrinses, high fluoride toothpaste)
- [ ] Management of hyposalivation
- [ ] Fissure sealants
- [ ] Sugar-free gum
- [ ] Calcium/phosphate-based strategies
- [ ] Xylitol-based strategies
- [ ] Antibacterial strategies
- [ ] pH neutralisation strategies

23. Does your dental school run a DENTAL THERAPY program?

- ◯ Yes
- ◯ No

Where and how is cariology taught – DENTAL THERAPY YEAR 1?

The following questions relate to DENTAL THERAPY YEAR 1

For each of the statements below, please enter a number in each column representing the number of LECTURES + SEMINARS + LABORATORY (PRECLINICAL SIMULATION) + CLINICAL sessions that each student receives in the various aspects of cariology curriculum in YEAR 1 DENTAL THERAPY.

* 24. How many hours are devoted to THEORETICAL teaching in Cariology?

Paediatric dentistry	
Operative/restorative/conservative dentistry	
Microbiology	
Biochemistry	
Oral pathology	
Preventive dentistry	
Community dentistry/dental public health	

25. How many sessions (of 2-3 hours duration) are devoted to PRE-CLINICAL NON-OPERATIVE experience in Cariology? (ie. behaviour management, motivational interviewing, fissure sealants)

Paediatric dentistry	
Operative/restorative/conservative dentistry	
Other	

26. How many sessions (of 2-3 hours duration) are devoted to PRE-CLINICAL OPERATIVE experience in Cariology? (ie. restorations)

Paediatric dentistry	
Operative/restorative/conservative dentistry	
Other	

27. How many sessions (of 2-3 hours duration) are devoted to CLINICAL NON-OPERATIVE experience in Cariology? (ie. behaviour management, motivational interviewing, fissure sealants, fluoride application)

Paediatric dentistry

Operative/restorative/conservative dentistry

Other

28. How many sessions (of 2-3 hours duration) are devoted to CLINICAL OPERATIVE experience in Cariology? (ie. treating patients)

Paediatric dentistry

Operative/restorative/conservative dentistry

Other

Where and how is cariology taught – DENTAL THERAPY YEAR 2?

The following questions relate to DENTAL THERAPY YEAR 2

For each of the statements below, please enter a number in each column representing the number of LECTURES + SEMINARS + LABORATORY (PRECLINICAL SIMULATION) + CLINICAL sessions that each student receives in the various aspects of cariology curriculum in YEAR 2 DENTAL THERAPY.

* 29. How many hours are devoted to THEORETICAL teaching in Cariology?

Paediatric dentistry

Operative/restorative/conservative dentistry

Microbiology

Biochemistry

Oral pathology

Preventive dentistry

Community dentistry/dental public health

30. How many sessions (of 2-3 hours duration) are devoted to PRE-CLINICAL NON-OPERATIVE experience in Cariology? (ie. behaviour management, motivational interviewing, fissure sealants)

Paediatric dentistry

Operative/restorative/conservative dentistry

Other

31. How many sessions (of 2-3 hours duration) are devoted to PRE-CLINICAL OPERATIVE experience in Cariology? (ie. restorations)

Paediatric dentistry

Operative/restorative/conservative dentistry

Other

32. How many sessions (of 2-3 hours duration) are devoted to CLINICAL NON-OPERATIVE experience in Cariology? (ie. behaviour management, motivational interviewing, fissure sealants, fluoride application)

Paediatric dentistry

Operative/restorative/conservative dentistry

Other

33. How many sessions (of 2-3 hours duration) are devoted to CLINICAL OPERATIVE experience in Cariology? (ie. treating patients)

Paediatric dentistry

Operative/restorative/conservative dentistry

Other

Where and how is cariology taught – DENTAL THERAPY YEAR 3?

The following questions relate to DENTAL THERAPY YEAR 3

For each of the statements below, please enter a number in each column representing the number of LECTURES + SEMINARS + LABORATORY (PRECLINICAL SIMULATION) + CLINICAL sessions that each student receives in the various aspects of cariology curriculum in YEAR 3 DENTAL THERAPY.

* 34. How many hours are devoted to THEORETICAL teaching in Cariology?

Paediatric dentistry

Operative/restorative/conservative dentistry

Microbiology

Biochemistry

Oral pathology

Preventive dentistry

Community dentistry/dental public health

35. How many sessions (of 2-3 hours duration) are devoted to PRE-CLINICAL NON-OPERATIVE experience in Cariology? (ie. behaviour management, motivational interviewing, fissure sealants)

Paediatric dentistry

Operative/restorative/conservative dentistry

Other

36. How many sessions (of 2-3 hours duration) are devoted to PRE-CLINICAL OPERATIVE experience in Cariology? (ie. restorations)

Paediatric dentistry

Operative/restorative/conservative dentistry

Other

37. How many sessions (of 2-3 hours duration) are devoted to CLINICAL NON-OPERATIVE experience in Cariology? (ie. behaviour management, motivational interviewing, fissure sealants, fluoride application)

Paediatric dentistry

Operative/restorative/conservative dentistry

Other

38. How many sessions (of 2-3 hours duration) are devoted to CLINICAL OPERATIVE experience in Cariology? (ie. treating patients)

Paediatric dentistry

Operative/restorative/conservative dentistry

Other

39. Does your school run a DENTISTRY program?

- ◯ Yes
- ◯ No

40. How long is your DENTISTRY program?

- 5 years
- 4 years (graduate entry)

Where and how is cariology taught – DENTISTRY YEAR 1?

The following questions relate to DENTISTRY YEAR 1

For each of the statements below, please enter a number in each column representing the number of LECTURES + SEMINARS + LABORATORY (PRECLINICAL SIMULATION) + CLINICAL sessions that each student receives in the various aspects of cariology curriculum in YEAR 1 DENTISTRY.

* 41. How many hours are devoted to THEORETICAL teaching in Cariology?

Paediatric dentistry

Operative/restorative/conservative dentistry

Microbiology

Biochemistry

Oral pathology

Preventive dentistry

Community dentistry/dental public health

42. How many sessions (of 2-3 hours duration) are devoted to PRE-CLINICAL NON-OPERATIVE experience in Cariology? (ie. behaviour management, motivational interviewing, fissure sealants)

Paediatric dentistry

Operative/restorative/conservative dentistry

Other

43. How many sessions (of 2-3 hours duration) are devoted to PRE-CLINICAL OPERATIVE experience in Cariology? (ie. restorations)

Paediatric dentistry

Operative/restorative/conservative dentistry

Other

44. How many sessions (of 2-3 hours duration) are devoted to CLINICAL NON-OPERATIVE experience in Cariology? (ie. behaviour management, motivational interviewing, fissure sealants, fluoride application)

Paediatric dentistry

Operative/restorative/conservative dentistry

Other

45. How many sessions (of 2-3 hours duration) are devoted to CLINICAL OPERATIVE experience in Cariology? (ie. treating patients)

Paediatric dentistry

Operative/restorative/conservative dentistry

Other

Where and how is cariology taught – DENTISTRY YEAR 2?

The following questions relate to DENTISTRY YEAR 2

For each of the statements below, please enter a number in each column representing the number of LECTURES + SEMINARS + LABORATORY (PRECLINICAL SIMULATION) + CLINICAL sessions that each student receives in the various aspects of cariology curriculum in YEAR 2 DENTISTRY.

* 46. How many hours are devoted to THEORETICAL teaching in Cariology?

Paediatric dentistry

Operative/restorative/conservative dentistry

Microbiology

Biochemistry

Oral pathology

Preventive dentistry

Community dentistry/dental public health

47. How many sessions (of 2-3 hours duration) are devoted to PRE-CLINICAL NON-OPERATIVE experience in Cariology? (ie. behaviour management, motivational interviewing, fissure sealants)

Paediatric dentistry

Operative/restorative/conservative dentistry

Other

48. How many sessions (of 2-3 hours duration) are devoted to PRE-CLINICAL OPERATIVE experience in Cariology? (ie. restorations)

Paediatric dentistry

Operative/restorative/conservative dentistry

Other

49. How many sessions (of 2-3 hours duration) are devoted to CLINICAL NON-OPERATIVE experience in Cariology? (ie. behaviour management, motivational interviewing, fissure sealants, fluoride application)

Paediatric dentistry	
Operative/restorative/conservative dentistry	
Other	

50. How many sessions (of 2-3 hours duration) are devoted to CLINICAL OPERATIVE experience in Cariology? (ie. treating patients)

Paediatric dentistry	
Operative/restorative/conservative dentistry	
Other	

Where and how is cariology taught – DENTISTRY YEAR 3?

The following questions relate to DENTISTRY YEAR 3

For each of the statements below, please enter a number in each column representing the number of LECTURES + SEMINARS + LABORATORY (PRECLINICAL SIMULATION) + CLINICAL sessions that each student receives in the various aspects of cariology curriculum in YEAR 3 DENTISTRY.

* 51. How many hours are devoted to THEORETICAL teaching in Cariology?

Paediatric dentistry

Operative/restorative/conservative dentistry

Microbiology

Biochemistry

Oral pathology

Preventive dentistry

Community dentistry/dental public health

52. How many sessions (of 2-3 hours duration) are devoted to PRE-CLINICAL NON-OPERATIVE experience in Cariology? (ie. behaviour management, motivational interviewing, fissure sealants)

Paediatric dentistry

Operative/restorative/conservative dentistry

Other

53. How many sessions (of 2-3 hours duration) are devoted to PRE-CLINICAL OPERATIVE experience in Cariology? (ie. restorations)

Paediatric dentistry

Operative/restorative/conservative dentistry

Other

54. How many sessions (of 2-3 hours duration) are devoted to CLINICAL NON-OPERATIVE experience in Cariology? (ie. behaviour management, motivational interviewing, fissure sealants, fluoride application)

Paediatric dentistry

Operative/restorative/conservative dentistry

Other

55. How many sessions (of 2-3 hours duration) are devoted to CLINICAL OPERATIVE experience in Cariology? (ie. treating patients)

Paediatric dentistry

Operative/restorative/conservative dentistry

Other

Where and how is cariology taught – DENTISTRY YEAR 4?

The following questions relate to DENTISTRY YEAR 4

For each of the statements below, please enter a number in each column representing the number of LECTURES + SEMINARS + LABORATORY (PRECLINICAL SIMULATION) + CLINICAL sessions that each student receives in the various aspects of cariology curriculum in YEAR 4 DENTISTRY.

* 56. How many hours are devoted to THEORETICAL teaching in Cariology?

Paediatric dentistry

Operative/restorative/conservative dentistry

Microbiology

Biochemistry

Oral pathology

Preventive dentistry

Community dentistry/dental public health

57. How many sessions (of 2-3 hours duration) are devoted to PRE-CLINICAL NON-OPERATIVE experience in Cariology? (ie. behaviour management, motivational interviewing, fissure sealants)

Paediatric dentistry

Operative/restorative/conservative dentistry

Other

58. How many sessions (of 2-3 hours duration) are devoted to PRE-CLINICAL OPERATIVE experience in Cariology? (ie. restorations)

Paediatric dentistry

Operative/restorative/conservative dentistry

Other

59. How many sessions (of 2-3 hours duration) are devoted to CLINICAL NON-OPERATIVE experience in Cariology? (ie. behaviour management, motivational interviewing, fissure sealants, fluoride application)

Paediatric dentistry

Operative/restorative/conservative dentistry

Other

60. How many sessions (of 2-3 hours duration) are devoted to CLINICAL OPERATIVE experience in Cariology? (ie. treating patients)

Paediatric dentistry

Operative/restorative/conservative dentistry

Other

Where and how is cariology taught – DENTISTRY YEAR 5?

The following questions relate to DENTISTRY YEAR 5

For each of the statements below, please enter a number in each column representing the number of LECTURES + SEMINARS + LABORATORY (PRECLINICAL SIMULATION) + CLINICAL sessions that each student receives in the various aspects of cariology curriculum in YEAR 5 DENTISTRY.

61. How many hours are devoted to THEORETICAL teaching in Cariology?

Paediatric dentistry

Operative/restorative/conservative dentistry

Microbiology

Biochemistry

Oral pathology

Preventive dentistry

Community dentistry/dental public health

62. How many sessions (of 2-3 hours duration) are devoted to PRE-CLINICAL NON-OPERATIVE experience in Cariology? (ie. behaviour management, motivational interviewing, fissure sealants)

Paediatric dentistry

Operative/restorative/conservative dentistry

Other

63. How many sessions (of 2-3 hours duration) are devoted to PRE-CLINICAL OPERATIVE experience in Cariology? (ie. restorations)

Paediatric dentistry

Operative/restorative/conservative dentistry

Other

64. How many sessions (of 2-3 hours duration) are devoted to CLINICAL NON-OPERATIVE experience in Cariology? (ie. behaviour management, motivational interviewing, fissure sealants, fluoride application)

Paediatric dentistry

Operative/restorative/conservative dentistry

Other

65. How many sessions (of 2-3 hours duration) are devoted to CLINICAL OPERATIVE experience in Cariology? (ie. treating patients)

Paediatric dentistry

Operative/restorative/conservative dentistry

Other

Non-Carious Tooth Loss

66. At your Dental School, is erosion of dental hard tissue also covered in the curriculum (either as part of Cariology or elsewhere)?

- Yes
- No

67. If Yes, which year is it taught?

Year

68. At your Dental School, are the defects of dental hard tissues such as abrasion or attrition also included in the curriculum?

- Yes
- No

69. If Yes, which year is it taught?

Year

Staff Training

70. For clinical teaching staff (whether part-time/casual or members of faculty), are there training or education programs in Cariology to make staff aware of current teaching philosophies/practices ensure consistent application of teaching philosophies in the clinic?

- ◯ Yes
- ◯ No

71. For clinical teaching staff (whether part-time/casual or members of faculty), are there calibration exercises to ensure consistency of teaching approaches in Cariology in the clinic?

- ◯ Yes
- ◯ No

72. Which other field(s) do you believe should be covered in a Cariology Curriculum?

- [ ] Anatomy and histology of dental hard tissue
- [ ] Aetiology of dental caries
- [ ] Aetiology of dental erosion
- [ ] Caries microbiology
- [ ] Diet and dental caries
- [ ] Saliva and salivary glands
- [ ] Dental plaque
- [ ] Behavioural sciences
- [ ] Special aspects of early childhood caries
- [ ] Descriptive and analytical epidemiology of dental caries
- [ ] Descriptive and analytical epidemiology of dental erosion
- [ ] Clinical and histological appearance of dental lesions
- [ ] Clinical and histological appearance of dental erosion
- [ ] Clinical detection of carious lesions
- [ ] Radiological tools for the detection of caries lesions
- [ ] Non-radiological tools for the detection of caries lesions
- [ ] Management of secondary caries
- [ ] Management of mouth dryness
- [ ] Remineralisation of dental hard tissue affected by caries
- [ ] Removal of dental hard tissue affected (affected dentine) by caries
- [ ] Atraumatic restorative treatment of carious defects in dental hard tissues
- [ ] Caries risk assessment
- [ ] Personal level caries prevention and monitoring
- [ ] Population level caries prevention
- [ ] Professional and individual plaque removal
- [ ] Chemical (chlorhexidine)and mechanical plaque removal
- [ ] Use of fissure sealants
- [ ] Role of fluorides in caries prevention
- [ ] Role of fluorides in prevention of dental erosion
- [ ] Aspects of fluoride toxicity

☐ Other (please specify)

73. Do you think there are any impediments to Cariology teaching within your dental program?

74. Do you have any other comments?

End of Survey

* 75. Thank-you for taking the time to complete this questionnaire.
Could you please enter your email address below in case we need to contact you for further information or clarification of your responses.

Confidentiality
Please note that the information provided to the Alliance for a Cavity Free Future (ACFF) – Australian Chapter in this questionnaire will form the basis of a report that will be published, however, all such material will be aggregated and responding schools will not be specifically identified in relation to any particular issue.
